# Massive Lymphocele Associated with Allograft Hydronephrosis: An Ultrasound Case Study 

**DOI:** 10.24908/pocus.v7iKidney.14992

**Published:** 2022-02-01

**Authors:** Harini Bejjanki, Kawther F Alquadan, Abhilash Koratala

**Affiliations:** 1 Division of Nephrology, Hypertension and Renal Transplantation, University of Florida Gainesville, Florida USA; 2 University of Texas MD Anderson Cancer Center Houston, Texas USA; 3 Division of Nephrology, Medical College of Wisconsin Milwaukee, Wisconsin USA

**Keywords:** renal transplant, allograft, lymphocele, hydronephrosis, point of care ultrasound

## Abstract

Lymphocele is a lymphocyte-rich fluid collection that results from disruption of lymphatics in the recipient during renal transplantation. While small collections resolve spontaneously, larger, symptomatic ones may cause obstructive nephropathy requiring percutaneous or laparoscopic drainage. Prompt diagnosis using bedside sonography may obviate the need for renal replacement therapy. Herein, we present a case of a 72-year-old kidney transplant recipient who developed allograft hydronephrosis secondary to compression by a lymphocele.

## Case File

**Figure 1:**
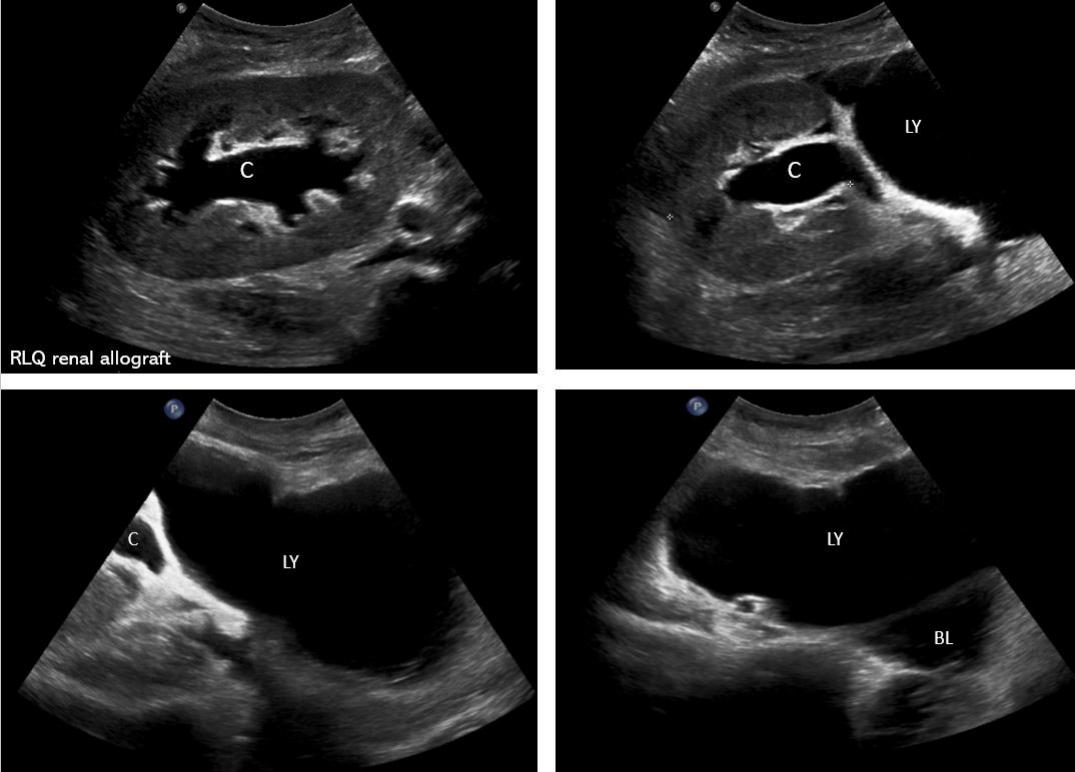
Sonographic images demonstrating a large hypoechoic collection (lymphocele) compressing the ureter and leading to hydronephrosis. Note the relationship between the lymphocele, renal collecting system, and the urinary bladder. RLQ = right lower quadrant; LY = lymphocele; C = renal collecting system; HY = hydronephrosis; BL = urinary bladder.

**Figure 2:**
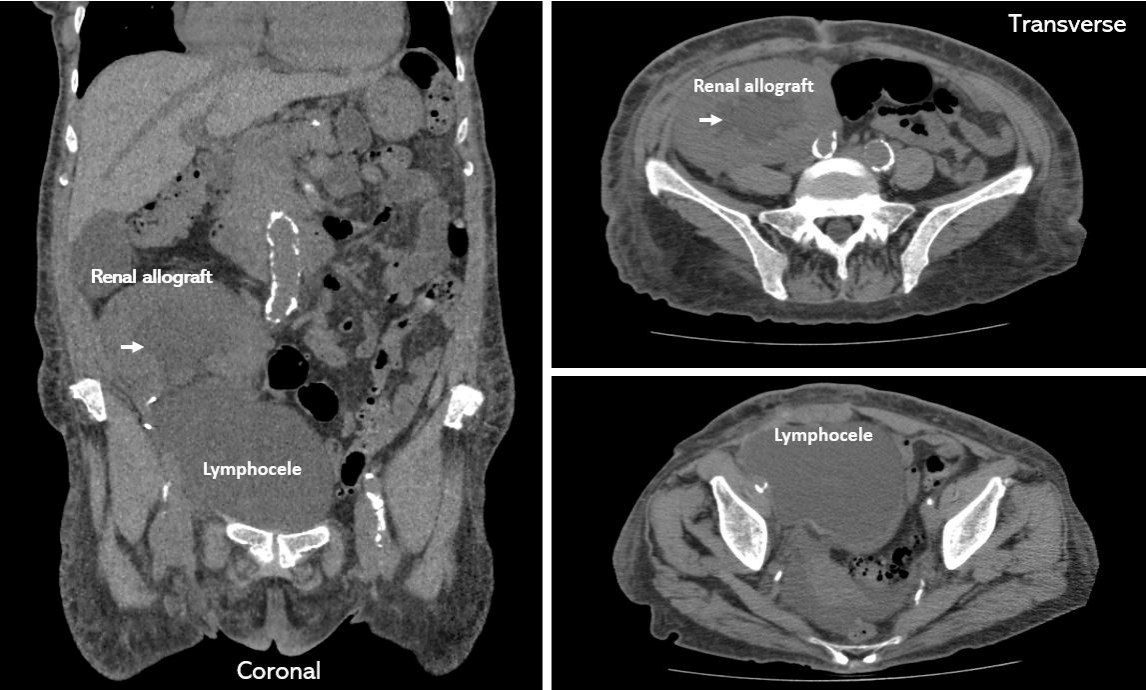
Computed tomography (CT) scan of the abdomen and pelvis without contrast demonstrating a large lymphocele occupying most of the pelvis. Arrows point to dilated renal collecting system/hydronephrosis.

**Figure 3:**
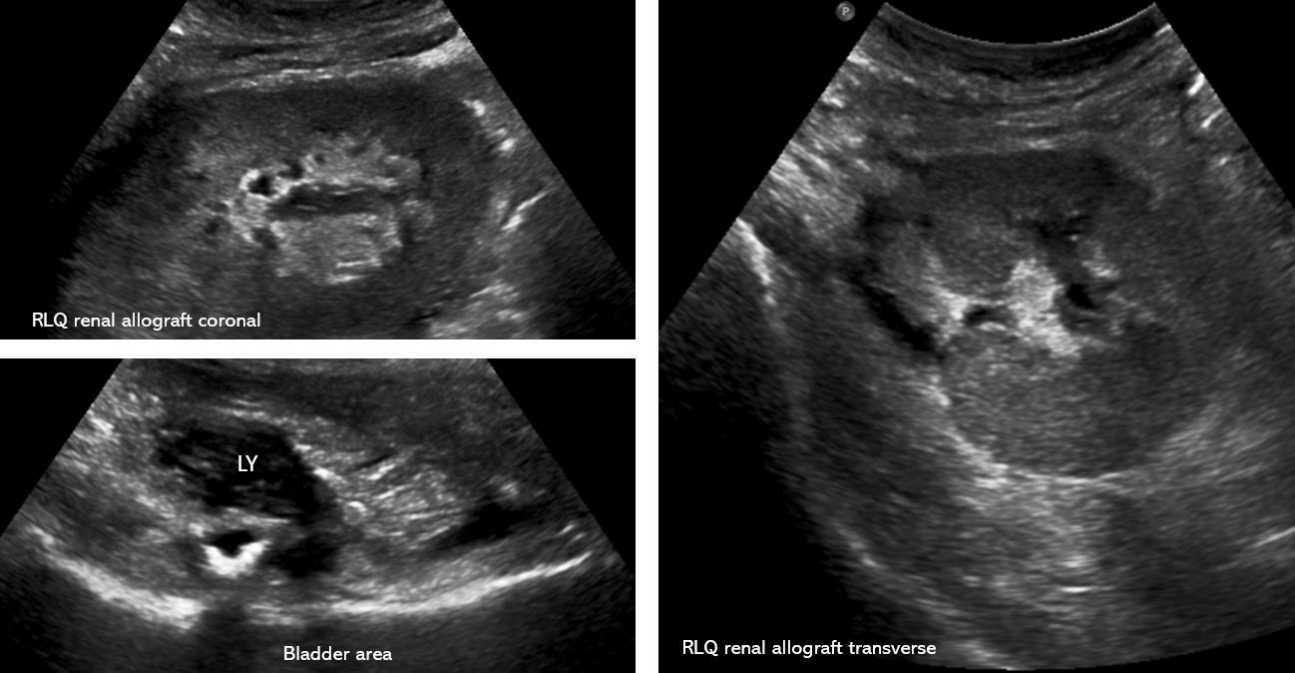
Repeat sonogram after the intervention demonstrating significant improvement in hydronephrosis and lymphocele.

A 72-year-old woman with a history of end-stage kidney disease underwent deceased donor kidney transplantation with a cold-ischemia time of 21 hours. Basiliximab was used for induction immunosuppression (IS), followed by maintenance IS consisting of mycophenolate mofetil, tacrolimus, and prednisone. Despite good urine output, her serum creatinine remained high ~4mg/dL and allograft biopsy demonstrated severe donor disease with arteriosclerosis, interstitial fibrosis, and tubular loss, though it excluded rejection. 20-days post-transplant, she developed oliguric renal failure requiring renal replacement therapy. A bedside renal sonogram demonstrated moderate hydronephrosis of the renal allograft secondary to compression of the transplanted ureter by a large anechoic fluid collection in the pelvis (Figure 1). Computed tomography (CT) scan confirmed the same (Figure 2) and the patient underwent ultrasoundguided drainage of approximately 450 cc fluid the next day with drain placement leading to improvement in urine output. Laboratory analysis of the fluid was consistent with lymphocele and a repeat sonogram showed significant improvement (Figure 3). One month later, the patient required a laparoscopic peritoneal window for drainage of the lymphocele into the peritoneal cavity, during which time, the drain was removed. The serum creatinine stabilized around 2.1mg/dL gradually over a month. 

A lymphocele typically occurs due to extravasation of lymph from the injured lymphatic vessels during preparation of the iliac vessels of the recipient and unligated lymphatics from renal hilum of the donor. These collections may develop in the early postoperative period or months following transplantation 1. The introduction of ultrasound evaluation has contributed to increasing the diagnosis and consequently the incidence of lymphocele especially of the asymptomatic ones. Before ultrasound examination, the incidence ranged from 0.6 to 18.1% 2. Small lymphoceles resolve spontaneously while the larger symptomatic ones may need percutaneous drainage or laparoscopy, with the later having least chance of recurrence 3. Sonographically, they appear as anechoic fluid collections around the allograft and must be distinguished from the adjacent urinary bladder and renal cysts. Rescanning after emptying the bladder aids in differentiating from the bladder. Renal cysts are typically well-circumscribed and connection with the renal parenchyma can be demonstrated. Moreover, history of kidney cysts in the donor kidney should be readily available, especially in the early post-transplant period. Some lymphoceles may also demonstrate internal septations and appear as multilocular cystic structures. 

In addition to rejection, structural abnormalities should be considered in renal transplant recipients with acute kidney injury, which are readily identifiable on a bedside sonographic examination in most cases. 

## Statement of Ethics

Informed consent obtained from the patient to publish this manuscript.

## Conflict of Interest 

The authors have declared that no conflict of interest exists. 

## Authorship 

All the authors have made substantial contribution to the preparation of this manuscript. HB: drafted the manuscript and performed literature search; KFA: attending physician on the case, directed patient care; AK: procured the images, critically reviewed, and revised the manuscript for important intellectual content. 
